# Novel Use of Calcimimetic Activity to Diagnose Primary Hyperparathyroidism in a Patient With Persistently Low-Normal Parathyroid Hormone Level

**DOI:** 10.7759/cureus.9360

**Published:** 2020-07-23

**Authors:** Sindhura Bandaru, Sukesh Manthri, Deepika Nallala, Chaitanya K Mamillapalli, Michael G Jakoby

**Affiliations:** 1 Internal Medicine, Southern Illinois University School of Medicine, Springfield, USA; 2 Oncology, East Tennessee State University, Johnson City, USA; 3 Endocrinology, Tower Health Medical Group, Wyomissing, USA; 4 Endocrinology, Springfield Clinic, Springfield, USA; 5 Endocrinology, Southern Illinois University School of Medicine, Springfield, USA

**Keywords:** primary hyperparathyroidism, parathyroid hormone, cinacalcet

## Abstract

Primary hyperparathyroidism (PHPT) is the most common etiology of hypercalcemia in the ambulatory setting and usually presents with an intact parathyroid hormone (PTH) level that is elevated or inappropriately near the upper limit of the laboratory reference range. However, PHPT with low-normal PTH level is reported in the peer-reviewed literature, and this atypical presentation may delay diagnosis of PHPT. We present a case of PHPT with persistently low-normal PTH level in which the PTH dependence of hypercalcemia was demonstrated by the response to treatment with the calcimimetic agent cinacalcet.

## Introduction

Primary hyperparathyroidism (PHPT) is the most common cause of hypercalcemia in the ambulatory setting, and prevalence in the United States approaches 1% [1]. There is a well-described female predominance of cases and a racial predilection for African Americans [2]. PHPT is caused by dysregulated secretion of parathyroid hormone (PTH) from one or more parathyroid glands [3]. PTH is unequivocally elevated in the majority of cases, though 5%-10% of patients have PTH levels in the laboratory reference range [4,5]. There are well-documented case reports of patients with PHPT and PTH levels that are low or low normal, and an uncharacteristically low PTH level may delay recognition of PHPT as parathyroid-independent etiologies of hypercalcemia such as malignancy typically suppress PTH to less than 20 pg/mL [6-10]. 

Cinacalcet is a calcimimetic agent that activates calcium-sensing receptors (CaSR) expressed by parathyroid glands, leading to reduction in PTH and serum calcium level in the majority of patients with PHPT [11]. The drug’s mechanism of action makes it possible to diagnose PTH-dependent hypercalcemia when PTH is low and other potential causes of hypercalcemia have been excluded. We present a case of PHPT with low PTH level in which a brief course of treatment with cinacalcet indicated the diagnosis of PHPT.

## Case presentation

A 57-year-old ambulatory male with a medical history notable only for hypertension and obesity was referred for evaluation of persistent hypercalcemia. Onset of hypercalcemia was in 2008, and the patient had multiple serum calcium measurements in the range of 10.5-11.5 mg/dL during the period 2008-2011 prior to referral (Table 1). The patient denied experiencing polyuria, episodes of nephrolithiasis, unusual abdominal pain, constipation, musculoskeletal pain, dyspnea, fatigue, night sweats, or unintentional weight loss. He was unaware of a family history of hypercalcemia. A regimen that included hydrochlorothiazide (HCTZ) had been prescribed for hypertension, though hypercalcemia persisted after HCTZ was stopped. Other serum chemistries, including serum creatinine and albumin level, were unremarkable. There were no significant findings on physical examination.

**Table 1:** Serum calcium and intact PTH levels ^†^St John’s Hospital and ^§^Memorial Medical Center, Springfield, IL; *Mayo Clinic, Rochester, MN; ^BC, Beckman Coulter, Brea, CA; ^#^treated with cinacalcet; multiply by 9.429 to convert from pM to pg/mL. Abbott, Chicago, IL; Roche, Basel, Switzerland. PTH, parathyroid hormone.

Date	Laboratory	Assay	Intact PTH (pM)	Intact PTH (pg/mL)	Reference range	Ca (mg/dL)	Reference range
March 2008	SJH^†^	Roche	2.8		1.2-7.0	10.9	8.4-10.5
March 2009	SJH	Roche	1.9		1.2-7.0	11.0	8.4-10.5
November 2009	MMC^§^	BC^		15	12-88	11.2	8.5-10.5
December 2009	MMC	BC		22	12-88	10.8	8.5-10.5
February 2010	SJH					10.9	8.4-10.5
August 2011	MMC					10.8	8.5-10.5
September 2012	SJH	Roche	2.5		1.2-7.0	10.7	8.4-10.5
October 2012	MMC	BC		18	12-88	10.9	8.5-10.5
November 2012	MMC	BC		24	12-88	11.0	8.5-10.5
July 2013	SJH	Abbott	2.3		0.9-7.7	11.8	8.4-10.2
September 2013	SJH					9.9^#^	8.4-10.2
October 2013	SJH	Abbott	1.9		0.9-7.7	11.0	8.4-10.2
December 2013	SJH	Abbott	1.9		0.9-7.7	9.4^#^	8.4-10.2
March 2014	Mayo*	Roche		15	15-65	-	

Multiple intact PTH levels in the range of 15-25 pg/mL were obtained (Table 1), prompting evaluation for PTH-independent causes of hypercalcemia. No abnormalities of parathyroid hormone related peptide (PTHrp), 25-hydroxyvitamin D, 1,25-dihydroxyvitamin D, thyroid-stimulating hormone, serum protein electrophoresis, and urine electrophoresis were detected (Table 2). The patient’s 24-hour urine calcium was >200 mg, excluding familial hypocalciuric hypercalcemia as a potential etiology of hypercalcemia. Serum phosphate level was at the lower end of the laboratory reference range. 

**Table 2:** Evaluation for PTH-independent etiologies of hypercalcemia PTH, parathyroid hormone; PTHrp, parathyroid hormone related peptide; TSH, thyroid-stimulating hormone.

Parameter	Result	Reference range/expected
PTHrp	<2.1 pM	0.0-4.0
1,25-dihydroxyvitamin D	45 pg/mL	15-75
25-hydroxyvitamin D	23 ng/mL	30-80
TSH	4.4 mIU/L	0.5-4.8
24-hour urine calcium	477 mg	<300
24-hour urine creatinine	2.4 g	0.7-2.2
Serum phosphorus	2.8 mg/dL	2.5-4.9
Serum protein electrophoresis	Normal pattern	-
Urine protein electrophoresis	No Bence-Jones proteins	-

Negative workup for PTH-independent hypercalcemia, coupled with hypercalciuria and low-normal serum phosphate level, lead to further evaluation for PHPT. Additional PTH measurements in the range of 15-25 pg/mL were obtained through the two local hospital laboratories and the Mayo Clinic reference laboratory (Table 1), and PTH level was unchanged on measurements at 1:3 and 1:6 dilutions of serum. Characteristics of the commercial intact PTH assays used to measure the patient’s serum PTH levels are summarized in Table 3. 

**Table 3:** Commercial intact parathyroid hormone assays Abbott, Chicago, IL; Beckman Coulter, Brea, CA; Roche, Basel, Switzerland.

Company	Capture mechanism	Reporter mechanism
Abbott	Goat antibody on microparticles	Goat antibody-acridinium conjugate
Beckman Coulter	Goat antibody on magnetic particles	Mouse antibody-alkaline phosphatase conjugate
Roche	Biotinylated mouse antibody/streptavidin-coated beads	Mouse antibody-ruthenium complex

**Figure 1:**
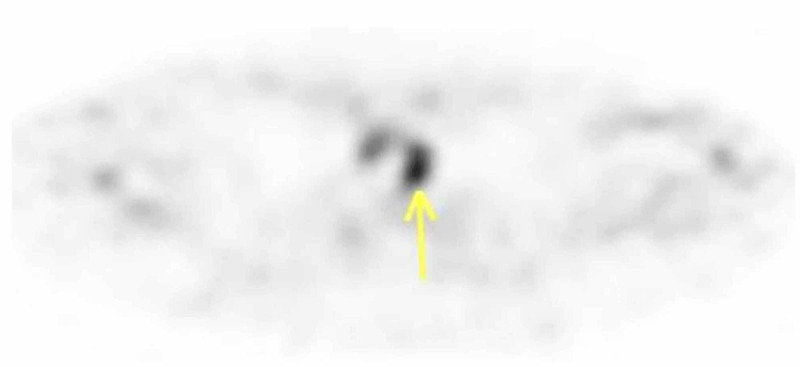
Parathyroid scan Parathyroid scintigraphy demonstrating persistent uptake of 99mTc sestamibi on 3-hour delayed images (yellow arrow) coinciding with a 6-mm mass posterior to the left lobe of the thyroid on single-photon emission computerized tomography (SPECT) felt to be consistent with a parathyroid adenoma.

Since the patient's degree of hypercalciuria was an indication for parathyroidectomy, parathyroid scintigraphy (Figure 1) was ordered. Persistent tracer uptake in the left lower neck that coincided with a 0.6-cm soft tissue density adjacent to the thyroid gland was demonstrated and felt to be consistent with a parathyroid adenoma. Unfortunately, an ultrasound-guided fine needle biopsy (FNB) of the mass failed to yield a specimen sufficient for cytological evaluation. 

**Figure 2:**
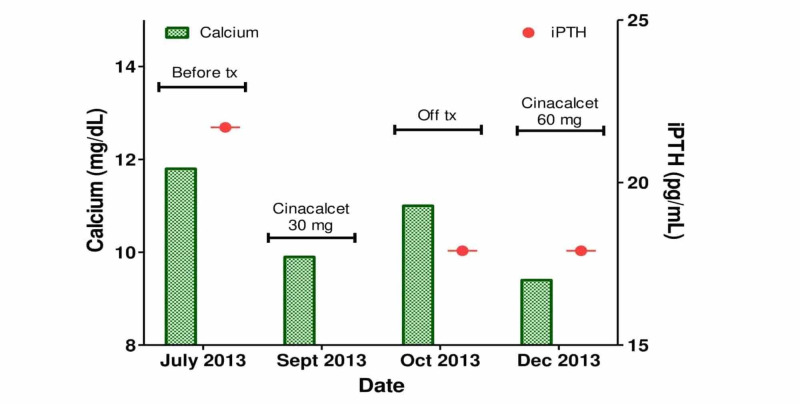
Serum calcium and intact PTH response to treatment with cinacalcet. Significant improvement in calcium level during treatment with the calcimimetic agent cinacalcet and rise in calcium level when the drug was stopped confirmed PTH-dependent hypercalcemia. Measured PTH level was unaffected by treatment with cinacalcet. PTH levels were multiplied by 9.429 to convert to pg/mL. PTH, parathyroid hormone; iPTH, intact parathyroid hormone.

Following the unsuccessful FNB, the patient agreed to treatment with cinacalcet to determine if hypercalcemia was PTH dependent. Results of the treatment trial are presented in Figure 2. Baseline serum calcium and intact PTH at the start of treatment with 30 mg cinacalcet twice daily were 11.8 mg/dL and 2.3 pM, respectively. Calcium level fell to 9.9 mg/dL during two weeks of treatment with cinacalcet and increased to 11.0 mg/dL one week after the drug was stopped. Cinacalcet was then restarted at 60 mg twice daily, and calcium level on active treatment improved to 9.4 mg/dL. Intact PTH level on the higher dose of cinacalcet remained stable (1.9 pM). 

The results of imaging and the trial of treatment with cinacalcet were felt to be diagnostic of PHPT due to a left-sided parathyroid adenoma. Parathyroid surgery was recommended due to the patient’s hypercalciuria. However, he declined surgery and was subsequently lost to follow-up.

## Discussion

The introduction of multichannel serum chemistry analyzers in the mid-1970s and highly sensitive two-site immunometric assays for PTH in the 1980s greatly facilitated early and accurate diagnosis of PHPT [12,13]. In the cohort of patients evaluated with the initial immunometric PTH assay, the lowest intact PTH level in the PHPT group was 50 pg/mL, and only 20 patients out of 271 (7.4%) with surgically confirmed parathyroid adenomas or hyperplasia had intact PTH levels in the laboratory reference range at time of diagnosis with PHPT [4,13]. The significant majority of patients with PHPT are distinguished by hypercalcemia and concurrent measurements of intact PTH that are either elevated or inappropriately high normal.

This case contributes to the literature establishing that parathyroid-dependent hypercalcemia can be diagnosed in individuals with inappropriately low or low-normal PTH levels and confirms an earlier report by Zwerling that a trial of cinacalcet can establish the PTH dependence of hypercalcemia [14]. There are several potential etiologies of PHPT with low or low-normal PTH level: (1) an inhibitor of the PTH immunometric assay, (2) hook effect from severe elevation of PTH level, (3) pulsatile secretion of PTH, (4) mutations of PTH that change an epitope recognized by an antibody in the two-site PTH assay but do not affect binding or activation of the PTH receptor, (5) posttranslational modification of PTH that affects assay recognition but not receptor activation, or (6) an unmeasured but biologically active PTH fragment [8]. The consistency of PTH measurements from three different commercial assays (Table 3) using different capture and reporter antibodies makes an inhibitor unlikely, the hook effect was excluded by the lack of an increase in PTH level on dilutions of the patient’s serum, and the persistence of hypercalcemia with low PTH over six years makes low values due to pulsatile secretion of PTH very unlikely. Since a low detectable level of PTH was present over many years, a biologically active but immunologically undetected PTH fragment is suspected as the cause of hypercalcemia in this patient’s case, though it was beyond the resources of this division to specifically characterize the molecule.

Since cinacalcet treats hypercalcemia by enhancing activity of the CaSR expressed on parathyroid tissue, it has the potential to distinguish parathyroid-dependent hypercalcemia in cases with unusually low PTH levels. The patient’s serum calcium response to starting, stopping, and then restarting cinacalcet presented in Figure 2 indicates parathyroid-dependent hypercalcemia in this case despite low and low-normal intact PTH measurements. The absence of a change in intact PTH level may provide indirect evidence of an immunologically undetected PTH fragment, though the reduction in PTH level during long-term treatment with cinacalcet is only about 5%-10% from baseline [15]. A left-sided parathyroid adenoma is strongly suggested by the results of parathyroid imaging (Figure 1), though a tissue diagnosis was not made due to inadequate tissue from an ultrasound-guided FNB and the patient’s decision to defer parathyroidectomy. Nevertheless, this patient’s experience extends on the previously published case of cinacalcet to distinguish parathyroid-dependent hypercalcemia with unusually low-normal PTH and further establishes the utility of a brief trial of cinacalcet to confirm the diagnosis of PHPT with low PTH level after non-parathyroid etiologies of hypercalcemia have been excluded [14].

## Conclusions

PHPT is the most common cause of hypercalcemia in the ambulatory setting, and the immunometric assay for intact PTH has improved the ease and accuracy of diagnosing PHPT. However, rare cases occur in which detected PTH is unusually low despite parathyroid-dependence of hypercalcemia. This case illustrates that after excluding PTH-independent etiologies of hypercalcemia, a brief trial of treatment with the calcimimetic agent cinacalcet is a useful diagnostic strategy to establish the parathyroid-dependence of hypercalcemia despite low PTH levels. 

## References

[1] Press DM, Siperstein AE, Berber E (2013). The prevalence of undiagnosed and unrecognized primary hyperparathyroidism: a population-based analysis from the electronic medical record. Surgery.

[2] Yeh MW, Ituarte PH, Zhou HC (2013). Incidence and prevalence of primary hyperparathyroidism in a racially mixed population. J Clin Endocrinol Metab.

[3] Bilezikian JP, Bandeira L, Khan A, Cusano NE (2018). Hyperparathyroidism. Lancet.

[4] Mischis-Troussard C, Goudet P, Verges B, Cougard P, Tavernier C, Maillefert J-F (2000). Primary hyperparathyroidism with normal serum intact parathyroid hormone levels. QJM.

[5] Wallace LB, Parikh RT, Ross LV (2011). The phenotype of primary hyperparathyroidism with normal parathyroid hormone levels: how low can parathyroid hormone go?. Surgery.

[6] Lafferty FW, Hamlin CR, Corrado KR, Arnold A, Shuck JM (2006). Primary hyperparathyroidism with low-normal, atypical serum parathyroid hormone as shown by discordant immunoassay curves. J Clin Endocrinol Metab.

[7] Benaderet AD, Burton AM, Clifton-Bigh R, Ashraf AP (2011). Primary hyperparathyroidism with low intact PTH levels in a 14-year-old girl. J Clin Endocrinol Metab.

[8] Au AYM, McDonald K, Gill A, Sywak M, Diamond T, Conigrave AD, Clifton-Bligh RJ (2008). PTH mutation with primary hyperparathyroidism and undetectable intact PTH. N Engl J Med.

[9] Bhadada SK, Cardenas M, Bhansali A (2008). Very low or undetectable intact parathyroid hormone levels in patients with surgically verified parathyroid adenomas. Clin Endocrinol.

[10] Rosner MH, Dalkin AC (2012). Onco-nephrology: the pathophysiology and treatment of malignancy-associated hypercalcemia. Clin J Am Soc Nephrol.

[11] Shoback DM, Bilezikian JP, Turner SA, McCary LC, Guo MD, Peacock M (2003). The calcimimetic cinacalcet normalizes serum calcium in subjects with primary hyperparathyroidism. J Clin Endocrinol Metab.

[12] Wermers RA, Khosla S, Atkinson EJ, Hodgson SF, O’Fallon WM, Melton 3rd LJ (1997). The rise and fall of primary hyperparathyroidism: a population-based study in Rochester, Minnesota, 1965-1992. Ann Intern Med.

[13] Nussbaum SR, Zahradnik RJ, Lavigne JR (1987). Highly sensitive two-site immunoradiometric assay of parathyrin, and its clinical utility in evaluating patients with hypercalcemia. Clin Chem.

[14] Zwerling HK (2012). A novel use of cinacalcet to distinguish primary hyperparathyroidism from hypercalcemia of malignancy in a patient with a low-normal parathyroid hormone concentration. Endocr Pract.

[15] Peacock M, Bilezikian JP, Klassen PS, Guo MD, Turner SA, Shoback D (2005). Cinacalcet hydrochloride maintains long-term normocalcemia in patients with primary hyperparathyroidism. J Clin Endocrinol Metab.

